# Cerebral glycerol during haemorrhagic shock in normal and raised intracranial pressure resuscitated with total REBOA: an experimental porcine study

**DOI:** 10.1007/s00068-026-03236-y

**Published:** 2026-06-15

**Authors:** S. Bader, A. Magnuson, C. Brorsson, G. Wallin, N. Löfgren, F. Löfgren, P-J. Blind, M. Öman, M. Olivecrona

**Affiliations:** 1 Department of Surgery, Faculty of Medicine and Health, Örebro University, Örebro, Sweden; 2 Clinical Epidemiology and Biostatistics, School of Medical Sciences, Faculty of Medicine and Health, Örebro University, Örebro, Sweden; 3 Department of Surgical and Perioperative Sciences, Anaesthesia and Intensive Care, Umeå University, Umeå, Sweden; 4 Department of Diagnostics and Intervention, Umeå University, Umeå, Sweden; 5 Department of Neurosurgery, Faculty of Medicine and Health, Örebro University, Örebro, Sweden

**Keywords:** Resuscitative Endovascular Balloon Occlusion of the Aorta, REBOA, Haemorrhagic shock, Cerebral microdialysis, Cerebral glycerol, Aorta occlusion

## Abstract

**Background:**

Haemorrhagic shock (HS) is frequently associated with secondary cerebral injury due to compromised cerebral perfusion. Resuscitative endovascular balloon occlusion of the aorta (REBOA) is an effective haemorrhage control strategy; however, concerns persist regarding its cerebral effects, particularly in the presence of elevated intracranial pressure (ICP). Cerebral microdialysis (CMD) derived glycerol (CGly) is a recognised marker of cellular membrane stress and injury.

**Objective:**

To investigate CGly dynamics during prolonged total REBOA (tREBOA) in HS and to compare cerebral cellular responses between animals with normal and elevated ICP.

**Methods:**

In this experimental porcine study, eighteen pigs were subjected to controlled HS and resuscitated with tREBOA for 90 min. Animals were allocated to either a normal ICP group (NICPG, *n* = 9) or an elevated ICP group (EICPG, *n* = 9). Continuous monitoring of proximal arterial pressure, ICP, and cerebral perfusion pressure was performed. CGly concentrations were measured using CMD throughout the experimental protocol.

**Results:**

tREBOA effectively restored proximal arterial pressure and cerebral perfusion pressure in both groups. CGly concentrations remained relatively stable during the haemorrhage phase and increased progressively during prolonged aortic occlusion. Importantly, CGly responses did not differ significantly between animals with normal and elevated ICP. The observed increases in CGly during prolonged occlusion were consistent with cellular membrane perturbation rather than irreversible neuronal injury.

**Conclusions:**

In this experimental model of HS, prolonged total REBOA restored macrocirculatory parameters but was associated with time-dependent increases in CGly. Elevated ICP was not associated with exacerbation of cerebral cellular membrane stress during total aortic occlusion under the conditions studied. These findings suggest that intracranial hypertension alone does not worsen cerebral cellular responses during tREBOA, while highlighting the importance of occlusion duration and the need for further studies to optimise cerebral protection during prolonged aortic occlusion.

## Introduction

Haemorrhagic shock (HS) remains one of the leading causes of preventable mortality in trauma patients and is frequently associated with secondary brain injury due to inadequate cerebral perfusion [1, 2]. Rapid loss of circulating blood volume results in systemic hypoperfusion and cellular hypoxia, conditions under which the brain is particularly vulnerable. Even brief periods of insufficient cerebral perfusion may lead to irreversible neuronal injury, secondary brain damage, and poor neurological outcome [3, 4]. In cases of severe haemorrhage, conventional resuscitative measures such as fluid administration, blood products, and vasopressor support may be insufficient to restore arterial pressure (AP) and cerebral perfusion pressure (CPP) to levels required for adequate cerebral oxygen delivery [5].

Aortic occlusion (AO) has historically been used as a resuscitative manoeuvre to redistribute blood flow to the heart and brain during profound haemorrhage [6]. More recently, Resuscitative Endovascular Balloon Occlusion of the Aorta (REBOA) has emerged as a less invasive alternative to open aortic cross-clamping via thoracotomy [7, 8]. By occluding the aorta, REBOA preserves the remaining circulating blood volume to the upper part of the body, increases proximal mean arterial pressure (pMAP), and reduces ongoing distal haemorrhage, thereby serving as a bridge to definitive haemostasis [9, 10]. While REBOA has demonstrated haemodynamic benefits and potential survival advantages, concerns persist regarding its effects on the brain.

The total occlusion of descending aorta (tREBOA) might raise pMAP to supraphysiological levels and potentially disturb cerebral autoregulation (CA) causing cerebral damage [11–17].

Consequently, applying total tREBOA in patients with traumatic brain injury (TBI) and elevated intracranial pressure (ICP) has been regarded by some authors as contraindicated, as theoretically, the supraphysiological pMAP caused by AO may exacerbate cerebral oedema and increase intracranial haemorrhage [16, 18–24].

Cerebral microdialysis (CMD) provides a unique method for monitoring biochemical events in brain extracellular fluid and allows early detection of metabolic and cellular disturbances [25]. CMD has been extensively used in both experimental and clinical neurocritical care settings to monitor cerebral metabolism in conditions such as TBI, subarachnoid haemorrhage, ischemia, and shock [26–28]. Among the metabolites measured by CMD, glycerol has received particular attention as a marker of cellular membrane integrity.

Glycerol is an end product of membrane phospholipid degradation and is normally present in the central nervous system primarily as a structural component of cell membranes [29, 30]. Under physiological conditions, glycerol is thought to have limited permeability across an intact blood brain barrier (BBB), and increases in cerebral glycerol (CGly) are therefore considered to reflect local membrane phospholipid breakdown rather than systemic metabolic changes [31, 32]. Experimental studies have demonstrated marked increases in (CGly) following ischemia, seizures, and TBI, correlating with activation of phospholipases, intracellular calcium influx, oxidative stress, and cellular membrane injury [33, 34]. Clinical CMD studies have similarly reported elevated CGly concentrations in patients with severe brain injury, often preceding rises in ICP and other signs of secondary deterioration [35, 36].

Despite the increasing clinical use of REBOA, the impact of prolonged total AO on cerebral cellular integrity during HS remains insufficiently characterised. Previous studies investigating cerebral effects of AO have largely focused on open cross-clamping during normovolaemic cardiac surgery, conditions that differ substantially from the hypovolaemic trauma setting [6, 37–39]. More recent experimental studies of REBOA as an HS resuscitative method in trauma with an intact brain have shown a haemodynamically beneficial effect on cerebral circulation [11, 15, 40–42]. However, only a few studies have investigated the haemodynamic effects of REBOA in HS associated with ICP elevation [11, 43]. Nevertheless, we did not identify any previous studies in the literature describing CMD derived glycerol during prolonged total AO in HS.

Accordingly, the primary aim of the present experimental study was to investigate cerebral cellular degradation during HS resuscitated with prolonged tREBOA by measuring interstitial CGly concentrations using CMD. A secondary aim was to compare CGly dynamics between animals with normal ICP and those with experimentally elevated ICP in model of extracerebral acute pressure loading model.

## Materials and methods

### Ethics

All experimental procedures were conducted in accordance with the Guide for the Care and Use of Laboratory Animals, National Research Council, Washington, DC, USA, 1996 and the Directive 2010/63/EU on the protection of animals used for scientific purposes. The research project was approved by the Animal Experimental Ethics Committee at Umeå University, Sweden (A 32–19).

### Anaesthesia and surgical preparation

For details about the experimental setup and protocol, see previous publication [11]. Briefly: After induction of general anaesthesia with ketamine, the anaesthesia was maintained with sodium pentobarbital, fentanyl, and midazolam throughout the experiment. The animals were tracheostomised and mechanically ventilated. Ventilation parameters were adjusted to maintain normocapnia, as verified by arterial blood gas analysis.

A 7 Fr arterial catheter was placed in the right carotid artery for continuous measurement of pMAP and blood sampling. A REBOA catheter was inserted in the right femoral artery and advanced to the descending thoracic aorta (Zone I).

An intraparenchymal catheter was used for ICP measurement (PSO-PTT, Sophysa, Orsay, France). The ICP catheter was connected to an ICP monitor (PSO-4000 Pressio 2, Sophysa, Orsay, France).

A cerebral microdialysis catheter (70 Brain Catheter, M Dialysis AB, Stockholm, Sweden) was inserted into the left frontal cerebral cortex, contralateral to the epidural balloon, to minimise local mechanical influence and to reflect regional cerebral metabolism and the catheter was perfused with fluid CNS (M dialysis Stockholm, Sweden) using a microdialysis pump (CMA 107; CMA/ Microdialysis Stockholm, Sweden) at a flow rate of 2 µL/min, following catheter insertion, a stabilization period was allowed before sampling commenced.

To create an elevated ICP in the elevated ICP group (EICPG) a Foley catheter was placed epidurally right side and successively filled with saline to create an elevated ICP of 25–30 mmHg.

### Experiment design and timeline (Fig. 1)

**Fig. 1:**
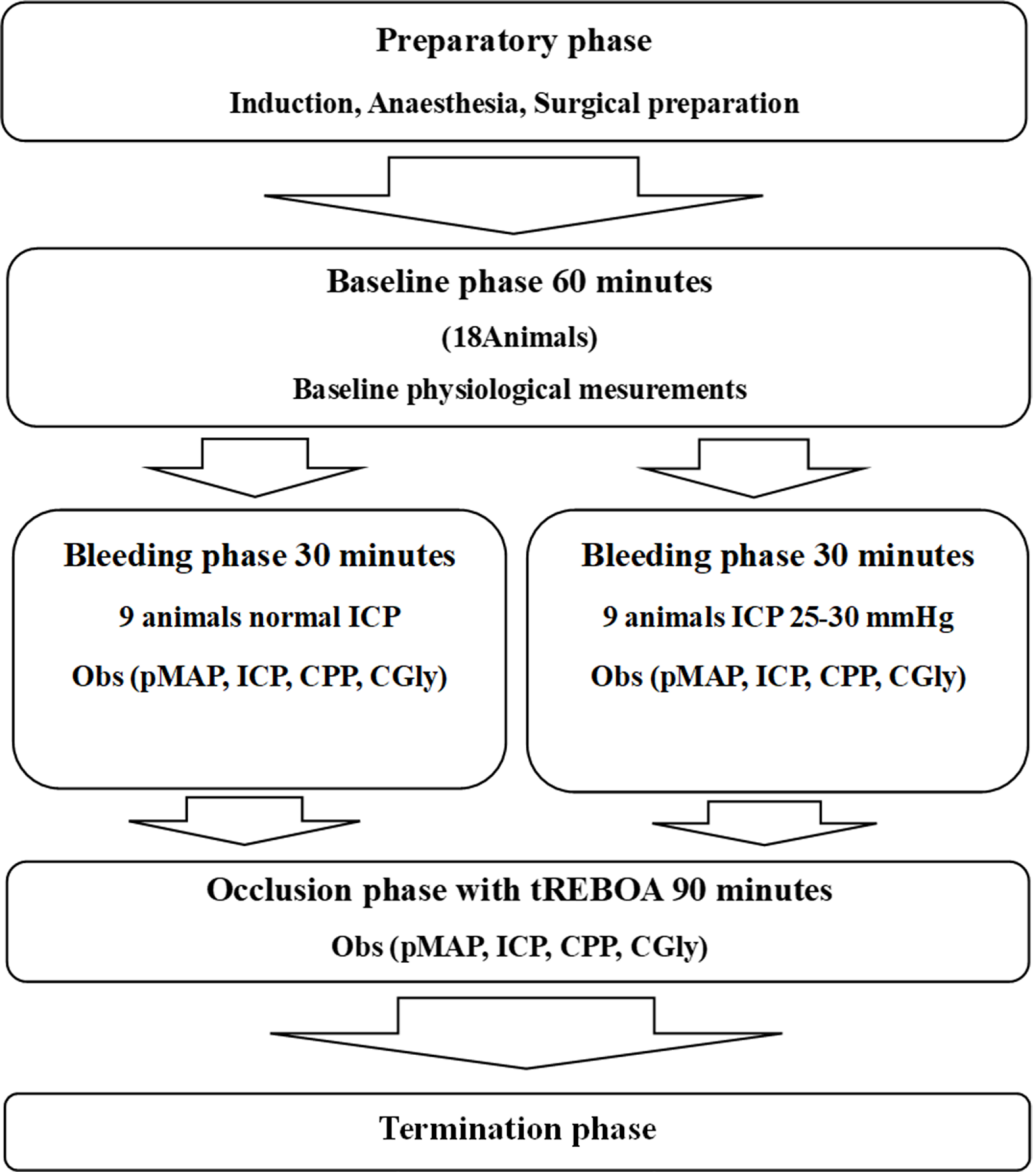
Experimental design and timeline

18 pigs were included in the study and divided in two groups. 9 with normal ICP (NICPG) and 9 with elevated ICP (EICPG). The experiment comprises 5 phases.

After completion of preparation and instrumentation, the animal was immobilised in supine position with 15° table inclination keeping the head and heart on the same level.

HS was induced by controlled withdrawal of approximately 40% of the estimated total blood volume through the arterial femoral catheter. Blood volume for each animal was estimated at 8% of the animal’s body weight and the blood was removed gradually over a period of 30 min to achieve and maintain a target mean arterial pressure (MAP) of approximately 40 mmHg. Adrenaline (Mylan, Canonsburg, Pennsylvania, USA) was given if needed to keep the MAP around 40 mmHg and to simulate the physiological stress response. In EICPG during this phase, the epidural catheter was inflated with saline to mimic an epidural haematoma.

Following completion of the haemorrhage phase, the balloon was inflated with saline until complete aortic occlusion (tREBOA), (T = 0 min). was confirmed by loss of femoral arterial waveform and abrupt increase in proximal arterial pressure Confirmation of adequate placement of the balloon above the diaphragmatic crus, in the thoracic descending aorta (zone 1), was obtained under tactile guidance. The balloon was then kept fully inflated for 90 min. After REBOA was inflated, 500 ml of 6% hydroxyethyl starch in sodium chloride (Voluven Fresenius Kabi, Homburg, Germany) was administered intravenously to mimic prehospital treatment of hypovolemia.

ICP, heart rate, proximal and distal AP, electrocardiography and pulse oximetry, were continuously monitored and manually recorded every five minutes throughout the experiment. Microdialysis CGly samples were collected at five-minute intervals throughout the experiment and were analysed using a bedside microdialysis analyser CMA 600 Analyser (CMA Microdialysis AB, Stockholm, Sweden. All data were stored digitally for subsequent analysis.

## Statistics

Statistical analysis was performed using a linear mixed-effects model to account for repeated measurements over time within individual animals. Time, experimental group and their interaction factor (time X group) were included as fixed effects, and individual animals were included as random effects. ICP and CGly were logarithmically transformed prior to analysis to achieve approximately normal distributed residuals. Sensitivity analyses were performed by excluding outliers with standardised residuals greater than 3 (in absolute values). Results are presented as means with corresponding 95% confidence intervals (CI). To reduce the risk of false-positive findings due to multiple testing between many time-points, a two-sided p-value of less than 0.01 was considered statistically significant. This conservative approach was selected to improve robustness, acknowledging the potential increased risk of type II error. Time-point 0 on experiment timeline is defined as the baseline for all variables. Statistical analyses were performed with STATA release 17 (StataCorp, College Station, TX).

## Results

### Study population

18 pigs (13 females, 5 males) with a mean body weight of 48.6 ± 6.9 kg were included in the final analysis. All animals completed the experimental protocol.

### Proximal mean arterial pressure (pMAP) (Fig. 2)

**Fig. 2:**
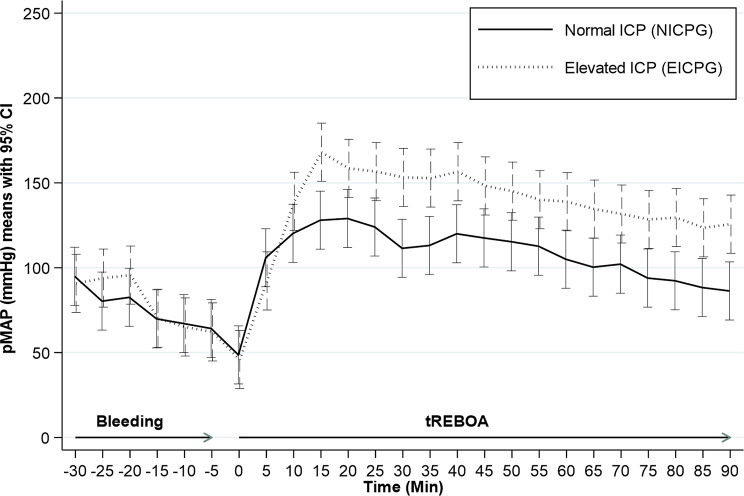
Proximal Mean Arterial Pressure (pMAP) vs. time

During the haemorrhage phase, pMAP decreased statistically significantly in both groups compared with baseline, from 95mmHg to 49 mmHg in NICPG (*P* < 0.01) and from 91 mmHg to 46 mmHg in EICPG (*P* < 0.01). Following REBOA inflation, pMAP increased rapidly and reached supraphysiological levels in both groups. From 15 min onward, pMAP was statistically significantly higher in the EICPG compared with the NICPG at multiple time points (*p* < 0.01), reflecting the interaction between elevated ICP and proximal arterial pressure (Fig. 2).

### Intracranial pressure (ICP) (Fig. 3)

**Fig. 3:**
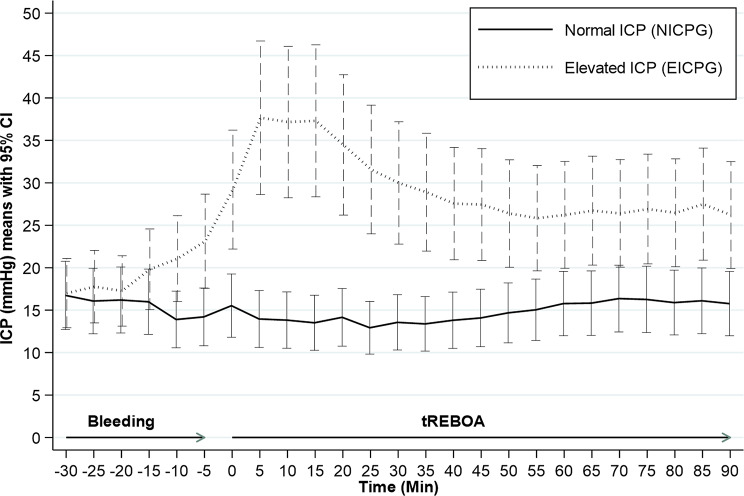
Intracranial pressure (ICP) vs. time

In the NICPG, the ICP was stable, and no statistically significant deviations from baseline were noted at any timepoint. In the EICPG, epidural balloon inflation resulted in a statistically significant increase in ICP prior to AO (to 29 mmHg *p* < 0.01), After peaking at 5 min the ICP gradually decreased remaining statistically significantly higher than in the NICPG throughout the entire occlusion period (Fig. 3).

### Cerebral perfusion pressure (CPP) (Fig. 4)

**Fig. 4:**
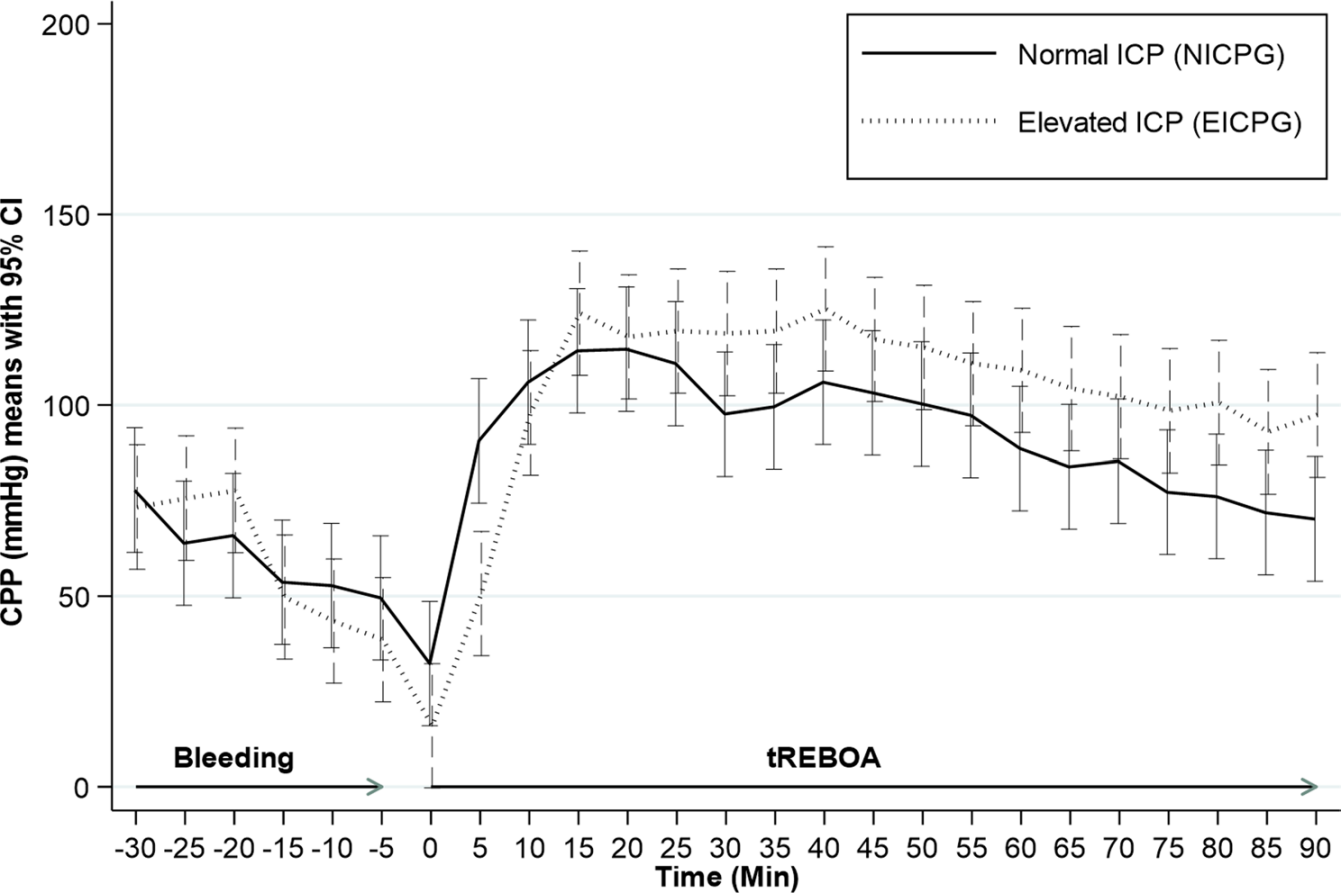
Cerebral perfusion pressure (CPP) vs. time

During the haemorrhage phase, CPP declined markedly in both groups in parallel with reductions in arterial pressure.

Following REBOA inflation, CPP increased rapidly in both groups. In the NICPG, CPP peaked at approximately 115 mmHg within 15–20 min post-occlusion and remained statistically significantly elevated compared with baseline throughout the occlusion period. In the EICPG, CPP increased to a maximum of approximately 124 mmHg at 15 min post-occlusion and remained statistically significantly elevated thereafter.

Between-group comparisons revealed no consistent significant differences in CPP during the occlusion phase (Fig. 4).

### Cerebral glycerol (CGly) (Fig. 5)

**Fig. 5:**
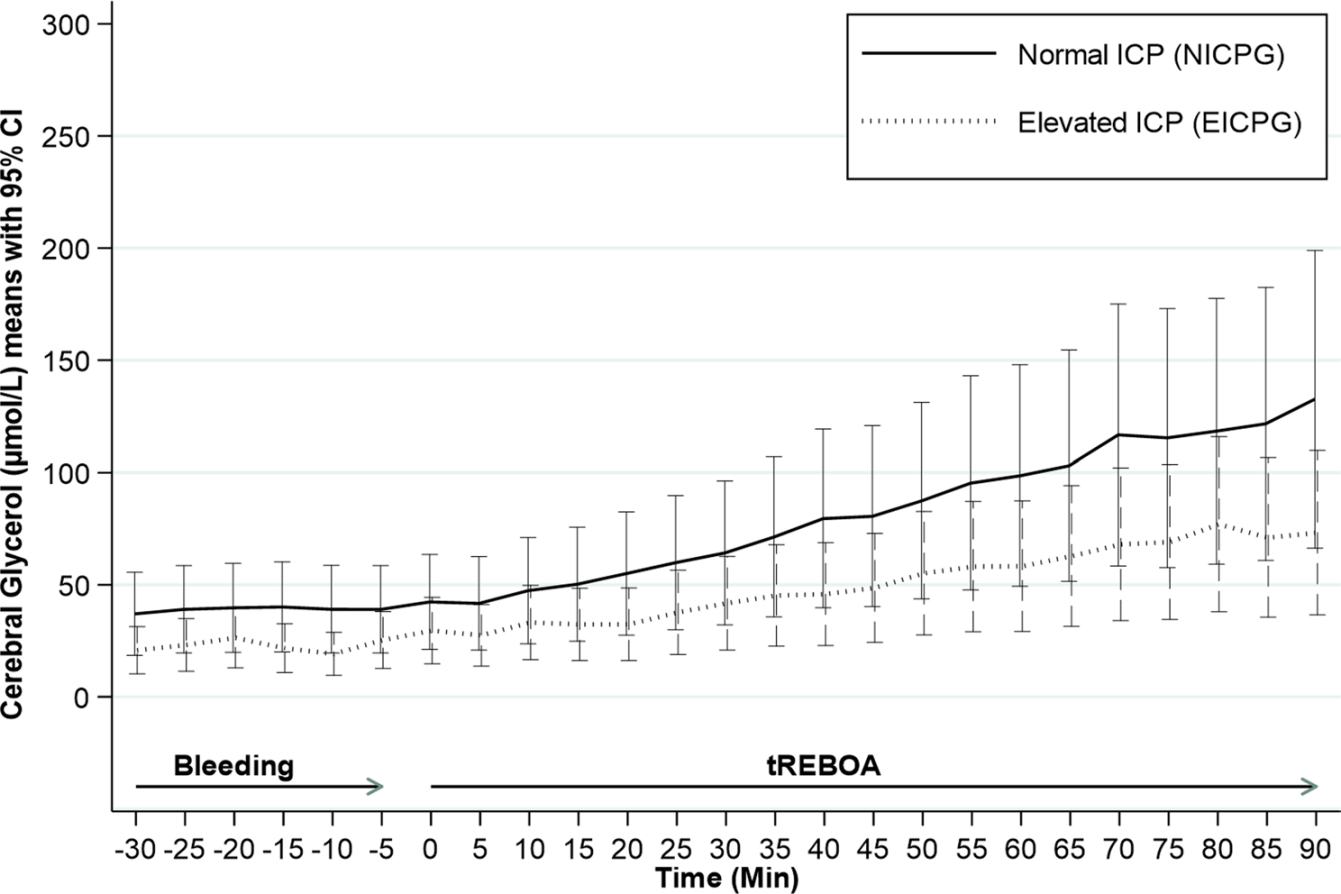
Cerebral Glycerol vs. time: Statistically significant increased (*p <*  0.01) 20 min after AO in NICPG and after 30 min in EICPG

Baseline CGly levels were comparable between groups, with a mean of 42 µmol/L in the NICPG and 30 µmol/L in the EICPG, with no statistically significant difference between groups at baseline (*p* > 0.01).

During the haemorrhage phase, CGly levels remained relatively stable in both groups, with no statistically significant changes compared with baseline.

Following REBOA inflation, CGly increased progressively over time in both groups. In the NICPG, a statistically significant increase compared with baseline was observed from 25 min post-occlusion onward (*p* < 0.01), with CGly levels continuing to rise throughout the occlusion period and reaching a mean of 132.62 µmol/L at 90 min (*p* < 0.01).

Similarly, in the EICPG, CGly increased statistically significantly following AO, with statistically significant elevations from 30 min post-occlusion onward (*p* < 0.01) compared to baseline. Peak CGly levels at 90 min reached a mean of 73 µmol/L.

Although CGly values were numerically higher in the NICPG throughout the occlusion phase, no statistically significant differences were observed between groups at individual time points or over time (time x group interaction, *p* > 0.01).

These findings indicate that CGly increased independently of ICP status, despite differences in absolute arterial pressure and ICP.

Despite consistently higher absolute CGly values in the NICPG compared with the EICPG, no statistically significant differences between groups were detected at individual timepoints during the occlusion period (Fig. 5).

## Discussion

The present study demonstrates a clear dissociation between macrocirculatory restoration and cerebral cellular integrity during prolonged tREBOA. Despite rapid and sustained increases in pMAP and CPP, C Gly concentrations increased progressively, suggesting ongoing membrane phospholipid degradation. This finding highlights that normalization or even supraphysiological elevation of CPP does not necessarily reflect adequate microcirculatory perfusion or cellular homeostasis.

The principal findings can be summarised as follows. First, tREBOA resulted in a progressive increase in CGly concentrations during the occlusion phase, despite the cerebral hyper perfusion in both experimental groups. Second, this increase was observed in animals with normal and as well as in those with elevated ICP, with not statistically significant between-group differences. Third, CGly concentrations remained stable during the haemorrhage phase and increased predominantly after AO, suggesting that the observed cellular response was closely related to the physiological consequences of tREBOA rather than HS alone.

Although elevated ICP was associated with lower CPP during the haemorrhage phase, CGly concentrations did not increase until after AO was initiated. This temporal dissociation indicates that impaired macrocirculatory parameters alone were insufficient to trigger detectable membrane phospholipid degradation under the conditions of this experiment [44–46]. Conversely, the sustained increase in CGly concentrations during the occlusion phase occurred despite supraphysiological CPP, highlighting a potential mismatch between restored macrocirculation and cerebral cellular homeostasis [47, 48].

Taken together, these findings suggest that prolonged tREBOA may be associated with subtle but progressive cerebral cell membrane phospholipid degradation that is not reliably reflected by conventional haemodynamic variables such as pMAP or CPP. Importantly, elevated ICP did not appear to exacerbate CGly release during tREBOA, challenging the assumption that intracranial hypertension per se necessarily increases the risk of cerebral cellular damage during AO [35, 36].

### Interpretation of cerebral glycerol increase despite restored CPP

Restoration of pMAP and CPP is a primary physiological objective during resuscitation from HS. In the present study, tREBOA rapidly increased both pMAP and CPP to supraphysiological levels in both experimental groups. Despite this apparent macrocirculatory restoration, CGly concentrations increased progressively during the occlusion phase, indicating ongoing disturbance of cerebral cellular integrity.

This dissociation between macrocirculatory parameters and cellular-level injury is a well-recognised phenomenon in neurocritical care. CPP reflects the pressure gradient driving cerebral blood flow (CBF) but does not directly account for microvascular distribution, capillary flow heterogeneity, or cellular metabolic demands [45, 46]. Experimental and clinical studies have demonstrated that cerebral tissue injury may evolve despite apparently adequate or even supraphysiological CPP, particularly when autoregulatory mechanisms are impaired or overwhelmed [48, 49].

Total AO induces abrupt proximal hypertension, which may exceed the upper limits of CA [12, 13, 15]. When CA fails, excessive perfusion pressure can result in microvascular shear stress, disruption of the BBB, and increased transcapillary filtration, promoting cerebral oedema and secondary cellular injury [12, 47]. In addition, prolonged AO is associated with ischemia reperfusion phenomena, oxidative stress, and inflammatory activation, all of which promote phospholipase activation and membrane phospholipid degradation [50–52].

Cerebral glycerol is a well-established marker of membrane breakdown. The absolute microdialysis concentrations are highly dependent on perfusion flow rate and probe recovery characteristics. The majority of the previous experimental and clinical cerebral glycerol studies have a microdialysis perfusion rate of 0.3 µL/min with normal reference value of 50–80 µmol/L. whereas the present study used microdialysis perfusion rate of 2 µL/min with normal reference value of 0–73 µmol/L, which limits direct comparison with previously published thresholds [30, 32, 33].

Marklund M et al. and Hillered L et al. have shown in their studies, with perfusion rate of 0.3 µL/min that moderate elevations (50–100 µmol/L) may reflect reversible cellular stress, whereas higher levels (> 100–150 µmol/L) are associated with more pronounced membrane degradation and potential structural injury [30, 33]. In the present study, glycerol levels reached values consistent with significant cellular stress, although the absence of histopathological correlation limits definitive conclusions regarding irreversibility. The progressive and sustained increase in CGly observed during the occlusion phase is therefore biologically plausible and consistent with established mechanisms of secondary brain injury [34]. Importantly, CGly concentrations remained within the physiological range during the haemorrhage phase and increased predominantly after AO was initiated, supporting the interpretation that the observed cellular response was related to tREBOA rather than HS alone [53, 54].

Notably, the increase in CGly was time dependent. In both experimental groups, CGly levels remained relatively stable during the early phase of AO and increased progressively with prolonged occlusion, becoming more pronounced after approximately 40–45 min [29, 55]. Marklund et al. in a TBI experimental model and Paschen et al. in a cerebral ischemia model indicate that CGly begins to rise 10–20 min after brain injury [30, 56].

### Normal vs. elevated intracranial pressure (NICPG vs. EICPG)

A central objective of the present study was to compare CGly dynamics during tREBOA in animals with normal and elevated ICP. Contrary to prevailing assumptions, elevated ICP was not associated with a more pronounced increase in CGly concentrations during AO. CGly levels tended to be higher in the NICPG, though no statistically significant differences were observed between groups at individual time points.

These findings challenge the assumption that intracranial hypertension per se necessarily exacerbates cerebral membrane perturbation during HS resuscitation with tREBOA [18, 19, 21].

According to the Monro Kellie doctrine, intracranial volume is fixed and composed of brain tissue, cerebral blood, and cerebrospinal fluid. Disruption of this balance increases ICP, which reduces CPP and may lead to cerebral ischaemia and neuronal injury [57–59].

Within the rigid skull, elevated ICP reduces the pressure gradient driving CBF. This effect is mediated through CPP [60]. As ICP rises and CPP falls, CA attempts to maintain CBF through vasodilation; however, high ICP can compress capillaries, increasing resistance and impairing oxygen exchange [11, 61, 62].

The reduced CGly concentration observed in the EICPG may be explained by two mechanisms. First, AO may have increased cerebral blood volume and pMAP sufficiently to improve cerebral perfusion. Second, elevated ICP can trigger the Cushing reflex, increasing pMAP to overcome high ICP and restore CPP [63]. ICP elevation may also limit acute cerebral hyper perfusion when CA is partially preserved [48, 64].

However, intracranial hypertension remains a major risk factor for secondary brain injury and poor neurological outcome and should not be interpreted as protective [65, 66]. The present findings therefore suggest that ICP alone was not the dominant determinant of glycerol release under the specific experimental conditions studied. Instead, global haemodynamic alterations, duration of AO, and microcirculatory and metabolic disturbances appear to play a more substantial role [35, 36].

### Clinical implications

The present findings have important implications for the clinical application of REBOA in HS. While tREBOA effectively restores pMAP and CPP, the time-dependent increase in CGly suggests that AO duration rather than ICP may represent the dominant determinant of cerebral cellular stress during total AO [9]. However, our model simulates epidural or extra cerebral pressure loading rather than true contusional brain injury, which may underestimate the degree of cerebral injury and limit direct clinical translation. On the other hand, this illustrates the acute rise in ICP short after an TBI, and so not the more slowly rising ICP which can follow from contusional injury.

These data support strategies aimed at minimising total occlusion time and exploring partial or titrated REBOA strategies, to avoid excessive proximal hypertension while maintaining adequate perfusion and to mitigate ischemia reperfusion injury [9, 15]. Furthermore, the dissociation between macrocirculatory restoration and cerebral cell membrane perturbation highlights the potential value of multimodal neuromonitoring, including CMD, in selected clinical settings [25, 27].

### Limitations

The present study has several limitations that should be acknowledged. First, the porcine model, while physiologically comparable to humans, does not fully replicate the complexity of polytrauma patients, including concomitant brain injury and cerebral contusion, coagulopathy, and systemic inflammatory responses. However, our experiment model represents acute epidural pressure model rather than a parenchymal traumatic brain injury. Second, the absence of other cerebral measurements like glucose, lactate, pyruvate, CBF, brain tissue oxygenation, BBB integrity and histopathological confirmation of neuronal injury. Third, 90 min of total aortic occlusion was longer than recommended in the clinical settings, and the observation period was limited and did not allow assessment of delayed or reperfusion-related injuries. Fourth, CMD provides focal biochemical and the absolute glycerol concentrations are influenced by methodological factors, including probe location, perfusion flow rate, anaesthetic regimen, and experimental conditions. For this reason, longitudinal trends rather than isolated values are considered more informative, and interpretation of absolute thresholds should be made with caution. Finally, Although CGly is a validated marker of membrane phospholipid degradation, it lacks specificity for irreversible neuronal death.

All these limitations restrict translational applicability and may underestimate the cerebral vulnerability observed in clinical settings. Future studies incorporating histological assessment and additional metabolic markers would strengthen the interpretation of cerebral cellular injury associated with prolonged aortic occlusion.

## Conclusion

In this experimental model of HS, prolonged tREBOA effectively restored pMAP and CPP. Importantly, CGly responses did not differ between animals with normal and elevated ICP, indicating that the presence of intracranial hypertension was not associated with an exacerbation of cerebral cellular membrane stress during total AO under the conditions studied. While increases in CGly were observed during prolonged AO, these findings should be interpreted as reflecting cellular membrane perturbation rather than irreversible neuronal injury. Overall, these findings suggest that elevated ICP alone may not necessarily exacerbate cerebral cellular membrane stress during tREBOA under the experimental conditions studied, and that tREBOA can serve as a bridge to further treatment. Our findings do not demonstrate absence of tissue injury, or long-term neurological recovery, although further studies are warranted to define optimal strategies for cerebral protection during prolonged AO.

## Data Availability

No datasets were generated or analysed during the current study.

## Abbreviations

AO: Aortic occlusion
AP: Arterial pressure
CA: Cerebral autoregulation
CBF: Cerebral Blood Flow
CGly: Cerebral glycerol
CMD: Cerebral microdialysis
CPP: Cerebral perfusion pressure
EICPG: Elevated Intracranial Pressure Group
HS: Haemorrhagic shock
ICP: Intracranial pressure
MAP: Mean arterial pressure
NICPG: Normal Intracranial Pressure Group
pMAP: Proximal mean arterial pressure
REBOA: Resuscitative Endovascular Balloon Occlusion of the Aorta
tREBOA: Total REBOA
TBI: Traumatic brain injury
